# Development and validation of a new analytical method for determination of linagliptin in bulk by visible spectrophotometer

**DOI:** 10.1038/s41598-023-31202-w

**Published:** 2023-03-11

**Authors:** Lujain Sahloul, Maisam Salami

**Affiliations:** Department of Analytical and Food Chemistry, Faculty of Pharmacy, Damascus University, Damascus, Syria

**Keywords:** Analytical chemistry, Characterization and analytical techniques

## Abstract

A simple, economical, and specific analytical method has been developed for determining and validating linagliptin (LNG) in bulk. This method is based on a condensation reaction between a primary amine in LNG and an aldehyde group in P-dimethylaminobenzaldehyde (PDAB) to form the yellow Schiff base with a wavelength of 407 nm. The optimum experimental conditions for the formulation of the colored complex have been studied. The optimum conditions were 1 mL of 5% w/v reagent solution with methanol and distilled water as a solvent for both PDAB, LNG respectively, also adding 2 mL of HCl as an acidic medium, heating to 70–75 °C on a water bath for 35 min. Furthermore, the stoichiometry of the reaction has been studied according to Job’s and Molar ratio method which was expressing 1:1 for LNG and PDAB. The researcher modified the method. The results show that the linearity in the concentration range (5–45 µg/mL) with correlation coefficient R^2^ = 0.9989 with percent recovery (99.46–100.8%) and RSD was less than 2%, LOD and LOQ 1.5815 − 4.7924 μg/mL respectively. This method can show high quality and there is no significant interference with excipients and in pharmaceutical forms. None of the studies showed the development of this method before.

## Introduction

**Figure 1:**
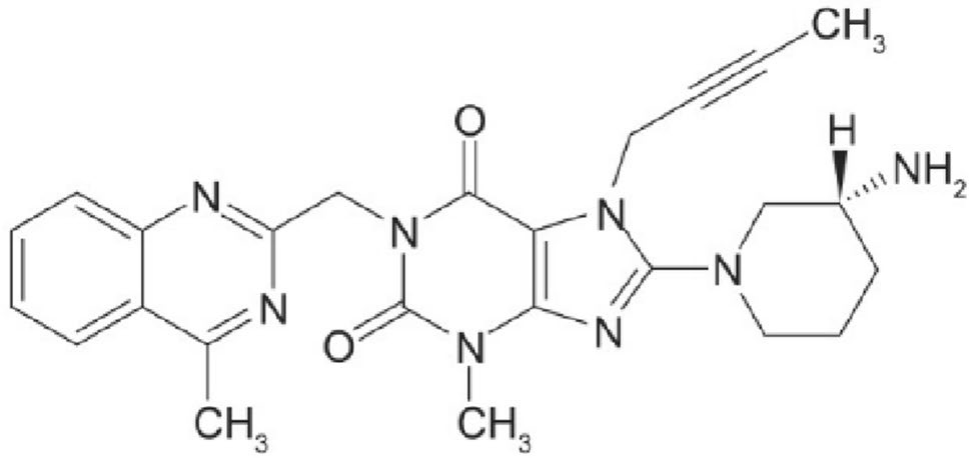
Chemical structure of linagliptin.

Linagliptin is a new dipeptidyl peptidase-4 (DPP-4) inhibitor, this enzyme is responsible for the downgrade of the incretin hormones glucagon-like peptide-1 (GLP-1) and glucose-dependent insulinotropic polypeptide (GIP). So this action increase the insulin level in the blood and the level of glucagon will be decreased^1^. It is used in combination with diet and exercise in the therapy of type 2 diabetes, either alone or in combination with other oral hypoglycemic agents (Empagliflozin, Metformin)^2^. The drug received FDA approval in May 2011^1^. As an oral antidiabetic agent, it has a xanthine-based structure, that may be a significant factor in the drug’s elimination half-life (more than 100 h). The long half-life of LNG may be more beneficial for patients who occasionally miss their doses of medication^1^. The chemical structure of LNG is 8-[(3R)-3-aminopiperidin-1-yl]-7-(but-2-ynyl-3-methyl)-1-[(4-methylquinazolin-2-yl)methyl]purine-2,6-dione.Molecular weight 472.5 g/mol (Fig. 1)^2^.

Chemical and Physical Properties of LNG: color and form: slightly hygroscopic, white to yellow solid. Melting Point: 190–195 °C. Solubility: In water, 3.33 mg/L at 25 °C; soluble in methanol; sparingly soluble in ethanol. Stability: It is stable if stored as directed; avoid strong oxidizing agents^2^.

Linagliptin isn’t available as a specific method for analyzing in British pharmacopeia (BP) or the United States Pharmacopeia (USP). The Review of Research Literature has shown several articles for the determination of LNG in pharmaceutical forms, including the spectrophotometer in UV^3–5^, also in VIS with chemical derivation using NQS (1,2-naphtho quinine 4-sulphonic acid sodium salt), vanillin^6^ and picric acid^7^ as a chromogenic reagents. Capillary Electrophoresis (CE)^8^, and with high-performance liquid chromatography (HPLC)^9–13^. Those methods are specificity and selectivity but they need more time, a lot of amount an expensive solvent and equipments.

While Vis Spectrophotometer is a simple, economical analytical method that it is used in multiple fields (Clinical biochemistry, chemistry…etc.). Also, it has high speed and doesn’t need extraction for detection of a small amount of material concentration compared to another method HPLC or CE. Due to LNG does not have a lot of chromophores, one chemical derivative method was used to develop a new spectrophotometer method for determination LNG in tablet products. This method needs less time-assuming, and has few solvents. Also, it has high precision and accuracy by using the derivation agent as PDAB that produces with the primary amine of LNG a yellow color of the complex having a maximum absorption at 407 nm.

## Materials and methods

The research method which is adopted for this paper was an experimental design that used an analytical approach to explore the research objectives.

### Instrumentation

- UV–Visible Spectrophotometer T80 + PG Instruments Ltd—England.
- Sartorius-Germany analytical balance.
- water bath.
- Calibrated glass pipettes.

### Materials and reagents

- Analytical grade Linagliptin, its purity was 99.25%, (Simson Pharma Limited—China),
- Methanol (99.9%, ACROS).
- Para-Dimethylaminobenzaldehyde (PDAB) 5% (w/v) (Scharlau-)
- Hydrochloride acid (HCl) 37% (SCP science)

### Preparation of linagliptin standard stock solution

A stock solution (1000 µg/mL): was weighed 50mg of LNG in 50 mL of distilled water.

### Preparation of linagliptin working solution

A working solution (100 µg/mL): 10 mL of stock solution is diluted to 100 mL with distilled water.

### Preparation of ρDAB 5% (w/v) solution

ρDAB 5% (w/v): 1.25 g was dissolved in 25 mL of methanol with good shaking, and was freshly prepared.

### Analytical procedure

Aliquots volume of working solution LNG were moved into series of 10 mL volumetric flasks to perform final concentrations of 5–45 ppm. To each flask was added 1 mL of ρDAB 5% (w/v) and 2 mL of HCl 37%, then to the water bath at 70–75° for 35 min after closed and shaking very well, after those flasks were cooled and diluted to 10 mL by distilled water. The maximum absorption of the yellow color was 407 nm upon the blank. The amount of linagliptin was calculated from the calibration curve.

This research was approved by the Damascus University Faculty of Pharmacy deanship.

### Ethics approval and consent to participate

Our study protocol was reviewed and approved by the Damascus University Faculty of Pharmacy, Damascus, Syria.

## Results and discussion

The analysis of the results that were gathered in the course of the research paper was sustained on four levels: the absorption spectra, Optimization of Reaction conditions, Stoichiometry of reaction and Validation of the developed method.

### Absorption spectra

**Figure 2:**
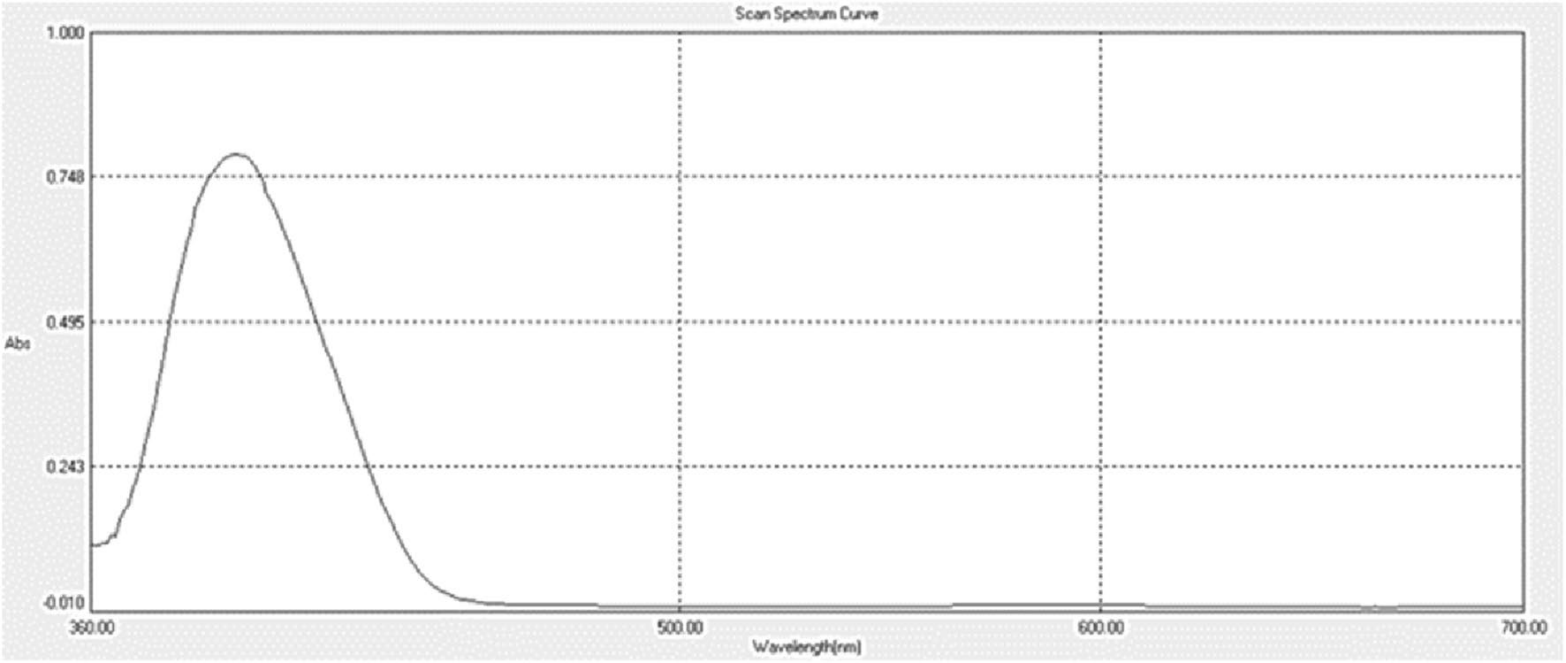
Absorption spectrum of the derivative product against a reagent blank (20 µg/mL LNG).

The absorption spectra of LNG (20 µg/mL) were recorded on a vis spectrophotometer in the wavelength region of 350–700 nm. which shows the absorption maxima curve at 407 nm in Fig. 2.

### Optimization of reaction conditions

#### Effect of diluting solvent

Many solvents such as distilled water, methanol, ethanol, and acetonitrile were chosen for solved LNG and PDAB as solvents for both LNG, and PDAB respectively, and as potential diluting media. Distilled water and methanol were found to be the optimum solvent for both LNG and PDAB respectively, and the highest absorbance values and the stability of the Schiff base formulated were obtained.

#### Effect of PDAB concentration

**Figure 3:**
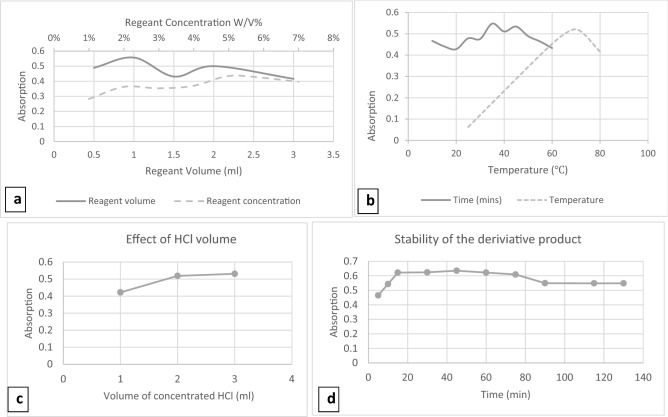
Optimization of Reaction conditions. (**a**) Effect of PDAB concentration (dashed line) and volume (dotted line) on the reaction of LNG with PDAB. (**b**) Effect of temperature (dotted line) and time (dashed line) on the reaction of LNG with PDAB. (**c**) Effect of HCl volume on the reaction between LNG (20 µg/mL) and PDAB 5%. (**d**) Stability of the reaction product.

The reaction between LNG and the increase of different PDAB concentrations (1–7% w/v) was studied. It was found that absorbance increases with increasing PDAB concentration and reaches its maximum value by using 5% w/v of reagent shown in Fig. 3a.

#### Effect of PDAB volume

The researcher studied the suitable of PDAB volume in the range of 0.5–3 mL, to find the suitable volume of PDAB reagent (5% w/v). So, the result had shown that the highest absorption intensity was achieved at a PDAB volume of 1 mL then it decreased (Fig. 3b).

#### Evaluation of HCl volume

The researcher tested different volumes of HCI 37% (1–3 mL), to select the appropriate acidic medium volume for LNG and PDAB reaction. The best results were obtained with 2 mL of HCl 37% (Fig. 3c).

#### Selection of temperature

To choose the best temperature which can achieve the objectives of the research, many different temperatures range (25, 60–65, 70–75, 80–85 °C) in the water bath was checked. Thus, it is possible to determine the best temperature range was 70–75 °C (Fig. 3b).

#### Select the optimum heating time

The effect of prolonged heating time on this reaction was controlled by monitoring the color development at different time intervals (10–60 min) at 70–75 °C. Maximum absorbance values were gained at 35 min (Fig. 3b).

#### Stability of the reaction product

For stability of the derivative product, it was tested by time intervals (5–130 min). The results showed that the derivative compound needs 15 min to reach the great absorption value and then the values remain constant for an hour (Fig. 3d).

### Stoichiometry of reaction

The quantitative reaction rate was recorded by Job’s Method of Continuous Variations and the Molar ratio method^14^.

Preparation of Linagliptin Standard Solution (2 × 10^–3^ mol/l) by weighted 94.5 mg of LNG in 100 mL of distilled water.

Preparation of ρDAB (2 × 10^–3^ mol/l) solution by dissolving 29.8 mg in 100 mL of methanol with good shaking, was freshly prepared.

**Figure 4:**
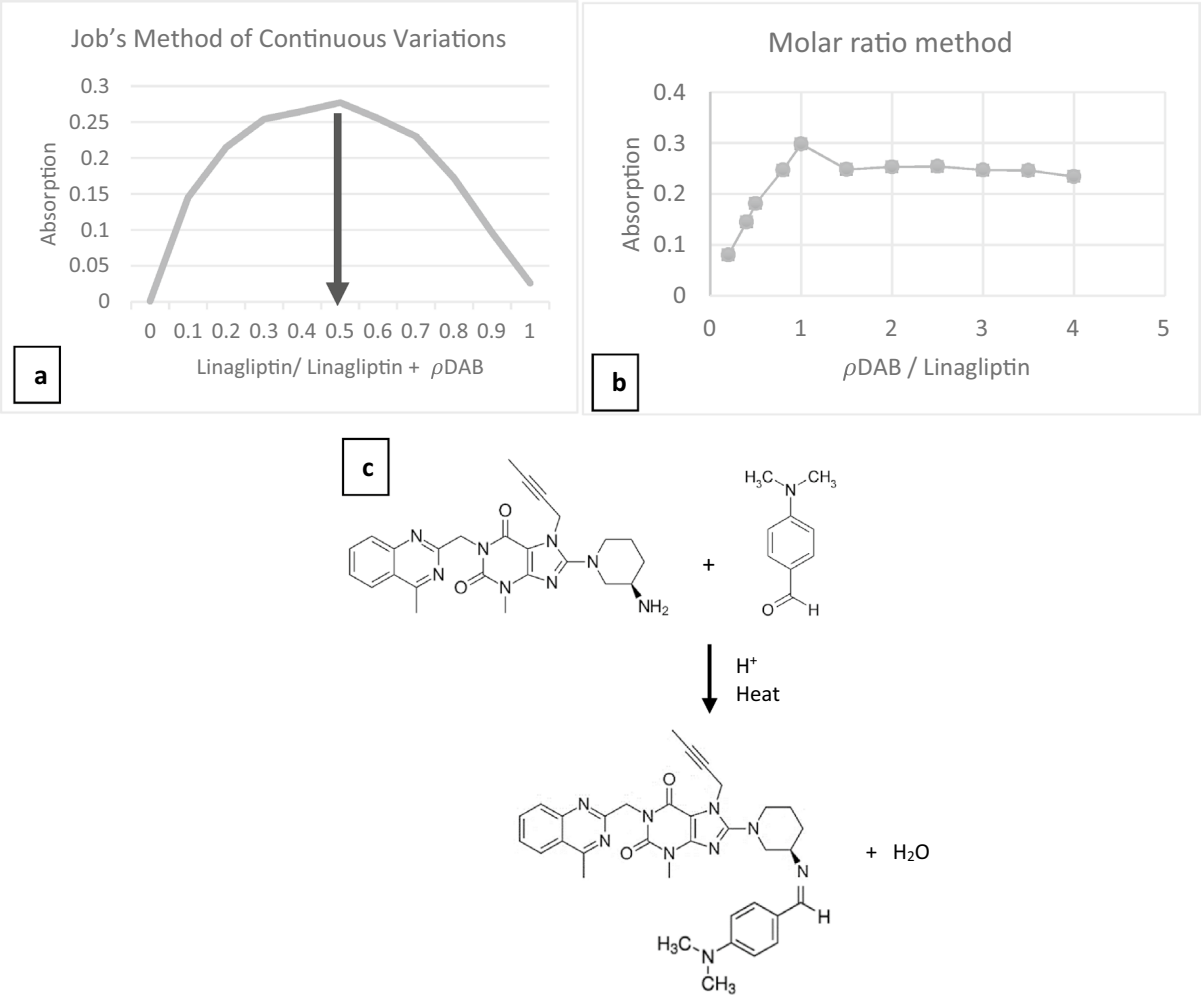
Stoichiometry of reaction LNG and PDAB. (**a**) Job’s method of continuous variations between LNG and PDAB. [LNG]: 2 × 10^−3^ M; [PDAB]: 2 × 10^−3^ M; [LNG] + [PDAB]: 4 mL + [HCl]: 2 mL. (**b**) Molar ratio method of stichometry of the reaction between LNG and PDAB. (**c**): Proposed reaction pathway between LNG and PDAB.

The continuous variation plot in Fig. 4a indicated that a molar fraction of 0.5 meant the ratio 1:1 of LNG: PDAB complex with 2 mL of 37% HCl for all flasks. While the Molar ratio plot in Fig. 4b, the highest absorbance was with LNG: PDAB complex ratio = 1. So, 1 mol of LNG interacts with 1 mol of PDAB.

The scheme of reaction between LNG and the reagent is shown in Fig. 4c.

### Validation of the developed method

The method was validated according to the procedures described in ICH guidelines, which included linearity, precision, accuracy quantitation limit, and detection limit^15^.

Linearity was evaluated after determining the optimum conditions, with Beer’s law used over the concentration, ranges 5–45 μg/mL, The calibration curve was formed by concentration versus absorbance, using linear regression analysis with an R^2^ value 0.9989 and regression equation was y = 0.0265x + 0.0602.

The limit of detection (LOD) and limit of quantitation (LOQ) were calculated according to the following formula:

$$ {\text{LOD }} = \frac{{3.3{ } \times {\text{SD}}}}{{\text{S}}},\;{\text{LOQ }} = \frac{{10{ } \times {\text{ SD}}}}{{\text{S}}} $$

where: SD is the standard deviation of the blank, and b is the slope. The LOD and LOQ were found to be 1.5815 and 4.7924 μg/mL, respectively (Table 1A)

### Precision and accuracy

The repeatability of the method was studied by measuring six replicate specimens of one concentration (20 µg/mL) of LNG within—day, with relative standard deviation RSD $\le \hspace{0.17em}2\%$ .

Furthermore, the intermediate precision was evaluated by analyzing three replicate solutions of LNG at three different concentrations (10–20–30 µg/mL) during the same day (intra-day) and over three successive days (inter-day), where no significant difference between intra- and inter-day precision values was observed and RSD % values were less than 2 (Table 1B).

**Table 1:** Validation of the developed method: (A) Parameters for the performance of the proposed method. (B) Accuracy and precision of the proposed method for the determination of LNG.

(A) Parameters for the performance of the proposed method			
Parameter	Value		
Measurement wavelength (nm)	407		
Linear range (μg/mL)	5–45		
Intercept (b)	0.0602		
Slope	0.0265		
Standard deviation of the blank	0.012751595		
Correlation coefficient (R2)	0.9989		
Limit of detection, LOD (μg/mL)	1.5815		
Limit of quantification, LOQ (μg/mL)	4.7924		
Molar absorbativity L mol^−1^ cm^−1^	19,500		
Sandell’s Sensitivity (µg/cm^−2^)	0.0242		

(B) Accuracy and precision of the proposed method for the determination of LNG			
Precision	Concentration (µg/mL)	Recovery %	RSD
Repeatability* (inter-day)	20	100.77	0.56
Intermediate precision** (intra-day)	10	99.66	0.648
	20	100.59	0.733
	30	100.7	0.455

Accuracy	Concentration (µg/mL)	Recovery %	Mean$\hspace{0.17em}\mp \hspace{0.17em}\mathrm{SD}$
(inter-day)**	10	99.42	100.156 ∓ 0.686
	20	100.78	
	30	100.27	

*Recovery value is mean of 6 replicates; RSD: relative standard deviation.

**Recovery values are mean of 3 replicates; SD: standard deviation.

About the accuracy that was checked by the percent mean recovery and RSD % between three concentrations of LNG (10–20–30 µg/mL). Table 1B.

### Sandell’s sensitivity (µg/cm^−2^)

$$   {\text{Sandell}}\;{\text{Sensitivity }}(\upmu {\text{g}}/{\text{cm}}^{{ - 2}} {\text{) = S}}\;{\text{per}}\;{\text{0}}{\text{.001}}\;{\text{absorbance}}\;{\text{unit,}}\;{\text{S  =  Molecular}}\;{\text{weight/}}\varepsilon.   $$

$$    {\text{Molar}}\;{\text{ absorbativity}}\;{\text{L}}\;{\text{mol}}^{{{{- 1}}}} \;{\text{cm}}^{{{{ - 1}}}} {\text{:according}}\;{\text{to}}\;{\text{Beer-Lambert}}\;{\text{law;}}\;{\text{A}} =  \varepsilon {\text{. b}}{\text{. M}}{\text{.}}    $$

### Specificity

The researcher had checked the interference of excipients that might be formed the pharmaceutical dosage by using this method, in order to determine the specificity of proposed method.

Sample was prepared by mixing mannitol 20 mg, maize starch 30 mg, pregelatinized starch 30 mg, copovidone 5 mg and magnesium stearate 4 mg^16^. These excipients were analyzed by proposed method. The results were referred good recovery 101.157% and RSD = 0.8387%. These means no interference between excipients and determination of LNG in pharmaceutical dosage by this derivative method.

### Robustness

The robustness of proposed method was examined, the results indicated that there were no bias in the experimental conditions (variation concentration of PDAB ∓ 0.2% (w/v) also volume of PDAB and HCl ∓ 0.2 mL, the heating time ∓ 2 min, measurement wavelength (nm) ∓ 2 nm, while others parameter were constant so RSD was less than 2%.

## Conclusion

Linagliptin is a new oral antidiabetic agent. LNG isn’t available a specific analytical method in pharmacopeia. LNG has many articles that aim to assay LNG by HPLC, this method needs expensive solvents and specific equipments. So, this paper was described a new, simple analytical method depending in condensation reaction between LNG with PDAB as a chemical reagent for determination LNG in bulk and pharmaceutical dosage. The conditions of optimal conditions of this analytical research were studied and it was found that distilled water and methanol were the best solvents for both LNG and PDAB, 1 mL of PDAB 5% as a derivative reagent with 2 mL of HCl 37% as an acidic medium with heating to 70–75 °C on a water bath for 35 min to form the yellow Schiff base with a wavelength at 407 nm and the stability of Schiff base formed for one hour. Validation of the proposed method was showed that this reaction has linearity 5–45 μg/mL, according to the correlation coefficient R^2^ = 0.9989 with percent recovery (99.46–100.8%) within an accepted criteria and RSD was less than 2%, so this proposed method has good accuracy and precision and high specificity.

Furthermore, the molar ratio of this reaction was selected between LNG and PDAB it was (1:1) complex depending on two methods the Job of continuous variations and molar ratio.

The proposed derivation method was characterized by the use of a PDAB reagent that is easy to apply and does not consume expensive materials compared to other analytical methods that require many devices and materials.

## Data Availability

The data can be made available upon reasonable request from the corresponding author.

## Abbreviations

PDAB: ρ-Dimethylaminobenzaldehyde
LNG: Linagliptin
GLP-1: Incretin hormones glucagon-like peptide-1
GIP: Glucose-dependent insulinotropic polypeptide
FDA: Food and Drug Administration
BP: British pharmacopeia
USP: United States Pharmacopeia
UV: Ultra-violet
VIS: Visible
CE: Capillary electrophoresis
HPLC: High-performance liquid chromatography
HCl: Hydrochloride acid
w/v %: Weigh/volume percent
ICH: International conference of harmonization
RSD: Relative standard deviation
SD: Standard deviation
